# Here I am, why don’t you answer me? Sensitivity to social responsiveness in domestic chicks

**DOI:** 10.1016/j.isci.2022.105863

**Published:** 2022-12-23

**Authors:** Maria Loconsole, Lucia Regolin

**Affiliations:** 1 Department of General Psychology, University of Padua, Padua, Italy; 2 Department of Biological and Experimental Psychology, School of Biological and Behavioural Sciences, Queen Mary University of London, London, UK

**Keywords:** Biological sciences, Ethology, Zoology

## Abstract

Newborn domestic chicks shortly exposed to a conspecific learn to recognize and prefer it over unfamiliar individuals. We assessed whether lack of physical contact or social feedback during familiarization affects affiliative preference, hypothesizing a crucial role of social responsiveness. Four-day-old chicks were tested for their preference between a familiar and an unfamiliar chick. In Exp. 1, we replicated the well-known preference for the familiar individual, even when (Exp. 2) a transparent glass prevented haptic interaction during familiarization. No preference was scored in Exp. 3, using a one-way glass, i.e., the subject could never be seen by its cagemate. The development of preferences toward a familiar but socially unresponsive cagemate was assessed by testing chicks twice (Exp. 4). While behaving at chance on day 2, birds showed a preference for the unfamiliar individual on day 4 of life. Our results highlight the importance of social interaction already in the first stages of life, irrespective of familiarity.

## Highlights

• Baby chick can recognize and prefer familiar over unfamiliar conspecifics
• Unresponsive social companions are not preferred to an unfamiliar conspecific
• Preference depends on subtle cognitive evaluations rather than familiarity
• Quality of social interaction is relevant already in the first stages of life

## Graphical abstract

**Figure:**
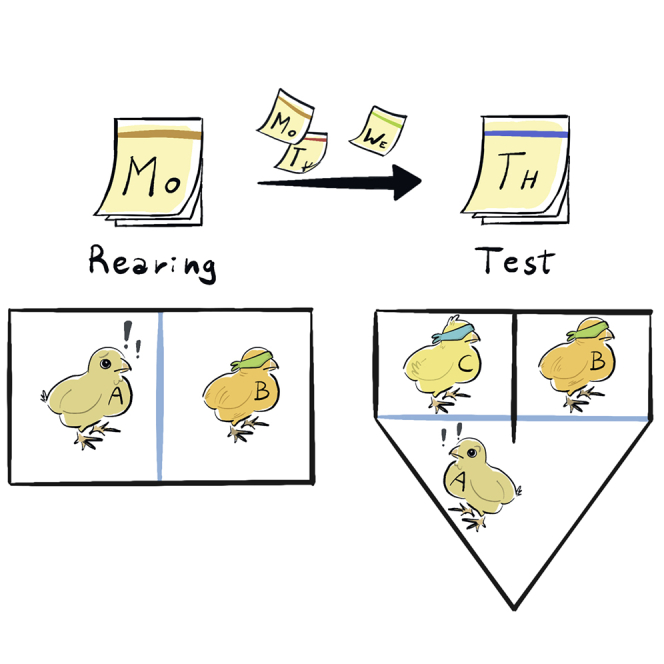

## Introduction

The domestic chick, as a precocial species, hatches predisposed to learn the characteristics of the social companions through filial imprinting and, following exposure, to develop a preference toward them.^1^^,^^2^^,^^3^ This process was finely described by Konrad Lorenz, and, as he suggested already in the title of his book “Here I am, where are you?”,^4^ imprinting is a learning process chiefly based on social interaction, possibly much more so than it has so far been recognized. Behavioral and neurobiological investigations provided a solid body of evidence to understand the biological basis of imprinting.^3^^,^^5^^,^^6^^,^^7^^,^^8^^,^^9^ According to current theories, imprinting lies upon two separate processes: a set of predispositions which orient the animal’s attention preferentially toward certain features (of color, pattern, motion, etc.); a subsequent learning phase in which all those characteristics are encoded as parts of one individual object.^10^^,^^11^^,^^12^ As a result of imprinting, the chick becomes familiarized with the object (e.g., a conspecific) experienced, which will be now preferred over novel, unfamiliar objects (individuals). Discrimination and preference for the familiar individual are considered a straightforward consequence of exposure. Absence of choice is instead ascribed to a failure in the imprinting process.^10^^,^^11^^,^^12^^,^^13^^,^^14^^,^^15^ Hence, if chicks have successfully imprinted on another chick, it implies that they will remember it, discriminate it from others, and exhibit a preference for it. In this process, the possibility to accommodate flexible decisions based on the quality of the interaction with the environment has not been considered. Under some circumstances, choice for the unfamiliar object has indeed been reported but argued to depend on explorative tendencies toward objects appearing as slightly novel with respect to the familiar one. Such preference would therefore depend on perceptual similarity rather than on a decision based on the fine processing of social communicative behaviors.^16^^,^^17^^,^^18^^,^^19^ The presence of sex-related differences, with males showing to prefer slight novelty more than female chicks,^17^^,^^19^ supports that this behavior is rooted in explorative tendencies, which differ in the two sexes.

We wondered whether chicks’ preference can be affected by the quality of the partner to which they are exposed, based on its behavior and on the reciprocity of the interaction. We tested whether chicks familiarized with a poorly responsive social partner would preferentially decide to approach a different (although novel) individual, to maximize the chances of engaging in a reciprocated social bonding. Such evidence would question the assumption that the preferential choice for the familiar individual is a sheer consequence of being exposed to such individual. Typically, the preference for the familiar individual is used as a proxy for the underlying mechanism of learning (i.e., the chick acquires relevant information about the conspecific) and memory (i.e., the chick can remember the features of the familiar individual at test). However, this assumption overlooks other subtler cues or cognitive processes that could affect chicks’ preference. It should be noted that, while exposure certainly allows the acquisition and subsequent retrieval of the characteristics of the familiar individual (i.e., memory and recognition), this may not always result in a preference for the familiar individual. Preferences could in fact be influenced by sophisticated decision-making processes based on fine elements as, in the case of the present study, the quality of the social interaction experienced during rearing.

In Exp. 1, we replicated the renowned result of filial imprinting following exposure, in which chicks that are reared together for some time manifest a preference for the familiar over an unfamiliar conspecific of the same age and sex. In Exp. 2, we tested the role of haptic contact in the development of affiliative preferences. It has been suggested that physical interaction is crucial for this as chicks reared together but separated by a meshed grid failed in discriminating between familiar and unfamiliar conspecifics.^20^ We hypothesized that other methodological factors may account for this result, and that chicks could instead develop a preference for the familiar conspecific even when deprived from physical interaction. Consistent with our hypothesis, we showed that preference for the familiar individual is expressed even when any haptic interaction among chicks was prevented during rearing using a transparent glass partition. This allowed us to test our second hypothesis on the role of social signals. In Exp. 3, chicks were reared in the same conditions as in Exp. 2 but they were separated by a one-way glass, so that chick A (subject) could see chick B (familiar conspecific), but B was not able to see A (i.e., B could only see its own reflection in the mirroring surface). This set-up allowed us to test subjects that had received identical exposure to the familiar conspecific as those in Exp. 2 but lacked any direct social feedback or interaction. Under this condition, chicks at test did not show a preference for the familiar individual. This null result supports our hypothesis according to which unsatisfactory social interaction might negatively affect affiliative preference. Yet, it does not allow us to disentangle whether the absence of choice resulted from lack of discrimination (e.g., if, during rearing, chicks were unable to learn the characteristics of their cagemate) or absence of preference (i.e., even though subjects could recognize the other chick, they were not motivated to preferentially approach it). To this aim, we reared a new group of chicks (Exp. 4) employing the same procedure as in Exp. 3 but testing them twice, on day 2 and on day 4 of life. Testing chicks at 48 h was aimed at assessing the possible presence of an initial preference (in line with studies on imprinting^2^^,^^21^) that might have disappeared after a prolonged negative experience (i.e., on day 4 of life). Surprisingly, we found that, while chicks already behaved at chance on day 2, they showed a clear preference for the unfamiliar chick on day 4. This result rules out the possibility of chicks being unable to discriminate familiar and unfamiliar conspecifics and supports the idea that reciprocated interaction is a crucial element in the development of social preferences following exposure.

## Results

We did not find an effect of overall time spent expressing a preference between experiments (X^2^ = 4.911, p = 0.297), suggesting that chicks were equally motivated to make a choice irrespective of the condition in which they were tested.

### Experiment 1: Standard rearing condition

**Figure 1:**
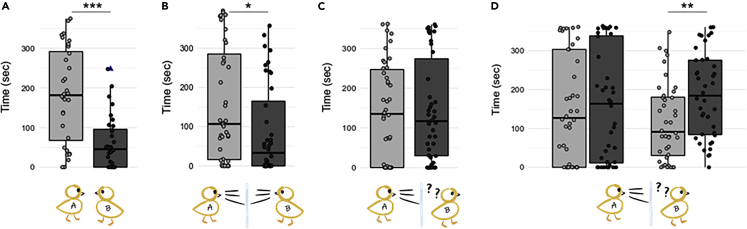
Results On the y axis, the time (in seconds) spent close to the familiar (in light gray) or the unfamiliar (in dark gray) conspecific. In the images: the experimental condition (from left to right, Exp. 1 to Exp. 4). “Chick A”: subject chick; “Chick B”: familiar chick. The boxplot shows the 25th percentile and the 75th percentile; the horizontal bar in the boxplot represents the median; the blue triangle in Exp. 1 indicates an outlier. Each dot represents the performance of a single subject. ∗p < 0.05; ∗∗p < 0.01; ∗∗∗p < 0.001. (A) In Exp. 1, chicks reared together preferred to rejoin the familiar conspecific and spent on average 2 min longer with it. (B) In Exp. 2, chicks separated by a transparent glass partition during rearing preferred to rejoin the familiar conspecific and spent on average 1 min longer with it. (C) In Exp. 3, chicks reared with a one-way mirror spent on average 5 s longer with the unfamiliar conspecific; this difference was not statistically significant. (D) In Exp. 4, chicks were reared with a one-way mirror and tested both on day 2 and day 4 of life. On day 2, they spent on average 10 s longer by the unfamiliar chick (not statistically significant). On day 4, chicks showed a significant preference for the unfamiliar conspecific and spent over 1 min longer closer to it.

In Exp. 1, we tested 32 female subjects. We found a significant effect of familiarity (X^2^ = 24.201, p < 0.0001). Overall, chicks spent longer time close to the familiar than the unfamiliar conspecific (estimated mean difference (familiar - unfamiliar) = 121 s, SE = 24, t = 5.02, p < 0.0001, Figure 1A). This preference emerged also as preferential first choice of the familiar chick (P(familiar) = 0.812, p < 0.001).

### Experiment 2: Rearing with a glass partition

In Exp. 2, we tested 40 female chicks. We found a significant effect of familiarity (X^2^ = 5.818, p = 0.016). Overall, chicks spent longer time close to the familiar than the unfamiliar chick (estimated mean difference (familiar - unfamiliar) = 70.8 s, SE = 29.3, t = 2.412, p = 0.021, Figure 1B). No preference appeared at the chicks’ very first choice (P(familiar) = 0.65, p = 0.081).

### Experiment 3: Rearing with a one-way glass

In Exp. 3, we tested 42 female subjects. Under this condition, we did not find any effect of familiarity (X^2^ = 0.027, p = 0.869). Chicks did not prefer any one of the two conspecifics over the other (estimated mean difference (familiar - unfamiliar) = −4.58 s, SE = 27.7, t = −0.165, p = 0.869, Figure 1C). Similarly, first choice was at chance level (P(familiar) = 0.476, p = 0.636).

### Experiment 4: Rearing with a one-way glass, developmental trajectory of the preference

In Exp. 4, we tested 40 female subjects both on day 2 and day 4 of life. It appeared that performance on day 2 of life and day 4 of life was not correlated (Pearson’s product-moment correlation, R = 0.07, t = 0.616, p = 0.54). For a more thorough inspection, we also run a linear model for addressing a possible effect of the day on the total time spent by the chicks close to either conspecific at test. In fact, the overall activity could be considered as a proxy of chicks’ motivation in engaging the task.^22^^,^^23^ We did not find an effect of the day on chicks’ overall activity (X^2^ = 0.077, p = 0.781), indicating that birds were similarly active in both tests, and as such we can assume a similar level of motivation/engagement. In light of these observations, we analyzed each test separately.

On day 2 of life, there was no effect of familiarity (X^2^ = 0.061, p = 0.805), and chicks did not prefer one of the two conspecifics over the other (estimated mean difference (familiar - unfamiliar) = −10.6 s, SE = 31.3, t = −0.34, p = 0.736, Figure 1D). Consistent with this, chicks did not show a preference on their first choice (P(familiar) = 0.425, p = 0.423).

On day 4 of life (i.e., the same age at which chicks were tested in all other experiments), we found a significant effect of the conspecific (X^2^ = 8.635, p = 0.003). However, contrary to Exp. 1 and 2, chicks displayed a clear preference for the unfamiliar individual: estimated mean difference (familiar - unfamiliar) = −68.8 s, SE = 23.4, t = −2.939, p = 0.006, Figure 1D). No preference was apparent in chicks’ first choice (P(familiar) = 0.5, p = 0.5).

## Discussion

Previous literature on affiliative preferences in baby chicks focused on imprinting learning and memory processes that eventually lead to acquiring and remembering the characteristics of a familiar individual to discriminate and prefer it over an unfamiliar individual.^2^^,^^3^^,^^4^^,^^6^^,^^12^^,^^21^ Here, we explored whether the preference for the familiar individual could be influenced by subtler aspects related to the quality of the social interaction experienced during exposure.

In Exp. 1, we showed that chicks that are reared together prefer to rejoin their social companion when separated, rather than approaching a novel individual of the same sex and age. This is in line with the prolific literature on filial imprinting also reporting a strong preference for the familiar over an unfamiliar conspecific in young chicks.^1^^,^^3^^,^^10^^,^^11^^,^^24^

In Exp. 2, we tested whether the absence of physical contact and haptic interaction could affect affiliative preferences. A previous study reported that chicks separated by a wire mesh did not show a preference among the familiar cagemate and unfamiliar chicks of the same age and sex.^20^ It was suggested that the opportunity to physically interact with conspecifics, as in pecking behavior, would be crucial to enable social learning and social discrimination, hence affiliative choices.^20^ We questioned this result, showing that chicks could successfully develop a preference for the familiar cagemate even when deprived from any physical interaction during rearing. Some methodological aspects may explain the difference in the obtained results. To answer the experimental hypothesis, Zajonc and colleagues (1975) tested two pairs of chicks simultaneously in an open field (45 × 45 cm) and scored the number of pecks directed toward the familiar or one of the two unfamiliar chicks as a proxy of familiarity, the hypothesis being that unfamiliar chicks would receive more pecks than the familiar one. While we agree on employing ecologic tasks in which the natural behavior of chicks can be observed, this paradigm may suffer from some confounding. The authors observed that most pecks they scored in the experiment were of exploratory nature (i.e., they did not differ from pecks directed to the environment, as the walls or the floors of the arena). Considering that even for the familiar subjects the test would represent the first moment of physical interaction, it is reasonable to assume that chicks could show exploratory pecks toward the familiar as well as the unfamiliar subjects, thus leading to a null result. Our Exp. 2 provides original evidence in favor of chicks being able to learn to recognize their familiar conspecifics even in the absence of physical contact.

In Exp. 3, we reared chicks separated by a one-way glass, for which one chick could see its companion but not be seen by this. Under these conditions, we expect the cagemate to not emit any social signal aimed at the subject chick. The cagemate could though emit signals directed at its own reflection, which would be in the same direction as the subject chick, or occasional tweets or pecks, yet none of these tuned to the behavior of the subject chick. The vocalization alone is unlikely to provide any valuable cue as all chicks were housed in cages in the same rearing room, where they could hear the calls of several other chicks out of the pairs. We consider the one-way glass as a condition of social impoverishment, as the chick’s social behavior is never reciprocated. Yet, at the same time, we maintained the same amount of visual exposure to the other chick as in Exp. 1 and Exp. 2, allowing to control for low-level perceptual variables. Under this condition, subjects did not show any preference in the free choice test. The free choice test is quite a common paradigm in studies on chicks’ social cognition, and birds usually display a clear preference toward the familiar individual.^3^^,^^4^^,^^14^^,^^24^^,^^25^ Moreover, there was no difference with respect to Exp. 1 and Exp. 2 in terms of overall time spent at test close to either conspecific. Thus, we excluded the possibility of chicks behaving at chance due to the testing set-up or to lack of motivation. In line with our initial hypothesis, learning might not have taken place in the absence of social feedback resulting in no preference for either conspecific. This would indicate how complex social cues can affect a predisposed response, such as the well-known familiarity preference in the baby chicks. However, as we reported an absence of choice, we cannot clarify its cause. On the one hand, it is possible that chicks failed to learn the characteristics of their cagemate during rearing, and thus could not discriminate it from the unfamiliar chick (hence, they approached both at random). Alternatively, it could be that chicks learned the characteristics of the familiar chick but, due to the prolonged negative social experience, did not express a preference for it, resulting in chicks behaving at chance level when tested on day 4.

To distinguish these two possibilities, we assessed a novel group of chicks reared in the same conditions as in Exp. 3 but tested twice, both at day 2 and day 4 of life (Exp. 4). We hypothesized a preference for the familiar chick may be found on day 2,^25^^,^^26^ and we expected absence of preference on day 4 (replicating Exp. 3). However, our hypothesis was not met. We found that chicks behaved at chance level already at the second day of life. Similar to what reported for Exp. 3, we deem this to be evidence of the crucial role of social experiences in shaping the predisposition to preferentially approach familiar conspecifics, suggesting that social feedback takes on great importance from the earliest stage of life. Interestingly, on day 4, we found a clear preference for the unfamiliar conspecific. This allows us to conclude that the absence of choice reported in Exp. 3 and in Exp. 4 on day 2 is best ascribed to absence of preference rather than lack of discrimination (i.e., to choose the unfamiliar chick, subjects must be able to recognize it as different from the familiar chick). A preference for the unfamiliar conspecific constitutes rather striking evidence of the importance of the social environment, and it could be interpreted as an exploratory behavior of a novel individual in the attempt to engage in a reciprocated social interaction. It might also constitute a strategy to avoid isolation and attempting to create better (i.e., more solid or long-lasting) social bonding. Conditions of social impoverishment or deprivation are associated with psychological (e.g., depression) and physical (e.g., undernourishment and compromised immune reactions) negative symptoms in several mammalian species.^27^^,^^28^^,^^29^^,^^30^ One of the most extreme examples can be found in a study showing how social isolation in the first year of life is associated with a significantly higher risk of premature death in monkeys.^31^ Similarly, it has been reported that in humans the lack of solid social relationships during infancy increases the risk of mental disorders and reduces the expected life span.^32^

It is yet to be addressed why 4-day-old chicks behaved differently in Exp. 3 and Exp. 4. The only difference between the two experiments is that chicks in Exp. 4 were tested both on day 2 and day 4 of life. In the first case, they behaved at chance level, similar to what was reported for 4-days-old chicks in Exp. 3. In the latter case, however, they preferred the unfamiliar conspecific. By comparing the overall times spent in the choosing areas, we found no difference between the three conditions, making it unlikely that the results are due to motivational factors or different exploratory tendencies. Additionally, in Exp. 4, the direction of choice on day 2 does not influence the subsequent choice on day 4 (i.e., there is no correlation between the two tests). The earlier test may simply act to familiarize the chicks with the arena. Familiarity with the setting may have played a key role in allowing the chicks to express their preference, without suffering from interfering cognitive processes related to the environmental changes (e.g., being in a novel situation would force chicks to allocate some resources to explore the environment and increase attentional responses). Further studies should be devoted to clarifying this point and better investigate the role of familiarity and different environmental pressures.

Overall, our study showed that baby chicks can evaluate the affiliative responses of their companions and, if these are defective, modify their social preference. This implies that the well-known preference for the familiar individual could hide a flexible and adaptive behavior which does not result uniquely from rigid predispositions and time of exposure as such but is finely shaped by the nature of the social interaction. This perspective better accounts for the importance of social interactions for this species, and the complex and sophisticated social life that characterizes individual from chicks to adults (for an exhaustive review, see^33^^,^^34^). Chicks are required to learn the characteristic of their social companions to discriminate them, but this does not necessarily result in a preference. This later is instead dependent on other complex variables, such as social factors. This opens to the study of what type of social feedback naive chicks are predisposed to expect, and their relevance in an evolutionary framework. We believe that these results should be considered for investigating the interaction between social cognition, learning, and innate predispositions in both social and non-social species. Importantly, we hope that our results provide relevant insights that could also reflect on current practices on animal welfare and husbandry.^35^

### Limitations of the study

We acknowledge some limitations of our study. In spite of being confident about the reliability of our paradigm (as supported by the results from Exp. 1 and Exp. 2), we could not completely rule out the role of environmental and task-related factors such as familiarity with the arena in Exp. 3 and Exp. 4. In fact, while this was not relevant when chicks were facing a considerably easy task (i.e., Exp. 1 and Exp. 2, where they showed a preference for the familiar individual), it may have interfered with subtler cognitive processes, such as those investigated in Exp. 3 and Exp. 4 (i.e., not only responding to familiarity but also evaluating social responsiveness). This could have masked or downsized a possibly stronger preference effect for the unfamiliar chick.

In order to register a clear response pattern, we selected only female chicks, as they are strongly motivated to rejoin the familiar companion when separated.^13^^,^^24^^,^^36^ Testing males with a similar paradigm would have led to difficulties in interpreting the behavior, as approach may not have been a consequence of affiliative but rather exploratory or even aggressive responses.^17^^,^^36^ It is yet to be addressed how social responsiveness is evaluated by male chicks. However, given the importance of social bonding for psycho-physiological well-being, we hypothesize a similar response both in male and female chicks.

## Availability of data and material

Raw data generated during the study are available in supplemental material S1.

## Ethics approval

The experiments complied with all applicable national and European laws concerning the use of animals in research and were examined and approved by the ethical committee of the University of Padua (Organismo Preposto al Benessere Animale - O.P.B.A.), N. Prot. 84260, N. Project 82/2021.

## STAR★Methods

### Key resources table

**Table:** 

REAGENT or RESOURCE	SOURCE	IDENTIFIER
**Experimental models: Organisms/strains**		

Domestic chicken (*Gallus gallus*) Broiler Ross 308	Incubatoio La Pellegrina, San Pietro in Gu (PD), Italy	https://en.aviagen.com/brands/ross/products/ross-308

**Software and algorithms**		

R: A Language and Environment for Statistical Computing	R core team	https://www.R-project.org/

### Resource availability

#### Lead contact

Further information and requests for resources should be directed to and will be fulfilled by the lead contact, Maria Loconsole (m.loconsole@qmul.ac.uk; mra.loconsole@gmail.com).

#### Materials availability

This study did not generate new unique reagents.

### Experimental model and subject details

This study used a total of 154 female domestic chicken (*Gallus gallus*) as the model organism. Chicks were tested on day 4 of life (Exp.1, Exp.2, Exp.3, and Exp.4), or on day 2 of life (Exp. 4). Fertilised Broiler Ross 308 eggs were purchased from a local hatchery (Incubatoio La Pellegrina, San Pietro in Gu, PD, Italy), and incubated (FIEM, MG 70/100 FAMILY) in a dark environment in the laboratory at controlled temperature (37.5°C) and humidity (50–60%) in the laboratory of Comparative Cognition at the University of Padua, Department of General Psychology.

Upon hatching, chicks were feather sexed to select the female subjects, which were randomly assigned to one of three possible rearing conditions (i.e., in pairs, separated by a transparent glass, or separated by a one-way glass). We decided to use female chicks as they are known to show a stronger motivation and a clearer response pattern in social tasks.^36^ Chicks were reared in pairs in standard metal cages (28 × 32 × 40 cm), with food and water *ad libitum*. Cages were illuminated from 7a.m. to 7p.m. light period and followed a 2- to 3- hours blocks of dark/light alternation from 7p.m. to 7a.m.

In Exp.1 (n = 32) chicks’ pairs could freely interact within the cage (Figure S1A). In Exp.2 (n = 40) a glass partition was introduced in the cage, so that chicks were physically isolated in the two resulting halves. Under this condition, chicks could see each other, but were deprived of any haptic information from the other individual (Figure S1B). In Exp.3 (n = 42) and Exp.4 (n = 40) chicks were divided by a partition similarly to Exp.2, with the only difference being that we employed a one-way glass instead of a transparent glass. The one-way glass allowed only one chick (i.e., the one that will be tested) to see its partner, but not to be seen. This rearing condition was employed to create a deprivation of both haptic and social interaction while still maintaining all visual information available to the subject chick (Figure S1C).

### Method details

The testing procedure and the apparatus were the same for all experiments (Figure S2). In Exp. 1, Exp. 2, and Exp. 3 chicks were tested on day 4 of life. In Exp. 4 the same chick was tested twice, on day 2, and again on day 4 of life. Chicks were individually tested in a separate room, maintained at the same temperature and humidity as the rearing room. The arena consisted of a triangular area created with white plastic sheets (60 × 30 × 55 cm). One vertex of the triangle represented the chick’s starting point. The animal was gently put in the vertex and restrained for few seconds by a glass partition. When the partition was lifted, the chick was free to move inside the arena. The basis of the triangle (opposite to the starting point) consisted of a one-way glass, that allowed the chick in the arena to see a rectangular area adjacent to the arena (60 × 15 cm). This area was further divided into two parts by a vertical opaque partition which extended in the triangular arena. In one of the two areas there was the familiar chick; in the other area there was an unfamiliar chick of same age and sex. The one-way mirror ensured that these two chicks could not see the tested chick, hence allowing us to control for any social cue that could have possibly biased the response at test. The two conspecifics were placed in the arena approximately 1 min before the testing chick, to allow them to settle in the new environment. Moreover, during test also the two conspecifics were monitored for signs of distress (e.g., emission of distress calls, hyper- or hypo-activation). In such cases, the experiment would have been immediately ceased and the chicks housed in social pairs and monitored in the rearing room (this was never the case in the present study). The vertical opaque partition served both to separate the two conspecifics (within the rectangular area in which they were confined) and to create two separate choice areas (30 × 15 cm each) in the testing arena, so that once the tested chick had approached one of the two conspecifics, it could not see the other one. The position of the familiar and unfamiliar chick was counterbalanced between subjects.

### Quantification and statistical analysis

The entire testing procedures were video recorded by a camera (Canon-Legria HF-R606) placed about 30 cm above the arena. The videos were analyzed offline using BORIS.^37^ For each subject we scored the time spent close to each of the two conspecifics. We considered a choice to be made when the chick passed the opaque partition with its head and at least 2/3 of the body, thus reaching a minimum distance of 5 cm from the chosen conspecific. We considered entering one of the two areas as a preference for the chick present on that side. Data were analyzed using R (R 4.2.0^38^). We adopted as a criterion for interpreting significant results p < 0.05. We employed a linear mixed model having as dependent variable the time spent in each choice area and as independent variable the conspecific (i.e., familiar or unfamiliar). Subjects were included in the model as the random effect. We tested the model fit using the DHARMa package.^39^ We then ran a post-hoc analysis with Bonferroni correction to determine the direction of the predictors, using the package emmeans.^40^ We ran a separate model to address whether the overall time that chicks spent by either stimulus changed between experiments, as an indicator of animals’ overall activity. We used this to evaluate animals’ motivation in engaging in the task. We analyzed chicks’ first approach at test employing a binomial test where 0 = choice of the unfamiliar chick and 1 = choice of the familiar chick. Graphs were generated using ggplot2.^41^

## Data Availability

• Data reported in this paper will be shared by the lead contact upon request. • This paper does not report original code. • Any additional information required to reanalyze the data reported in this paper is available from the lead contact upon request.
