# Supplementary material for: Here I am, why don’t you answer me? Sensitivity to social responsiveness in domestic chicks
Source: iScience. 2022 Dec 23;26(1):105863. doi: 10.1016/j.isci.2022.105863 (PMC9826874; doi:10.1016/j.isci.2022.105863)
Supplement: Document S1. Figures S1 — and S2 [file mmc1.pdf]

iScience, Volume *26*

Supplemental information

Here I am, why don't you answer me? Sensitivity to social responsiveness in domestic chicks

Maria Loconsole and Lucia Regolin

FIGURE S1. The rearing conditions for each Experiment, Related to STAR Methods

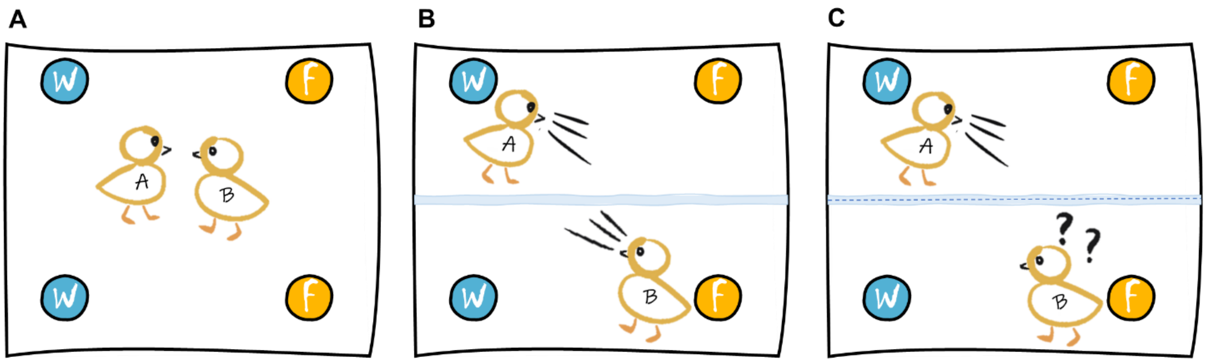

Fig. S1 Rearing conditions in each experiment: W and F: position of the water/food jars within the cage (W = water; F = food). A. Chicks could freely interact within the cage (Exp. 1). B. Chicks were separated in the cage by a glass partition. The glass prevented haptic interaction, but the two chicks could see each other and socially interact by visual cues (Exp. 2). C. Chicks were separated in the cage by a one-way glass. The glass worked so that chick A could still see chick B, but not vice-versa (i.e., chick B could see its own reflection in the mirror). Chick B was not expected to emit any visual social signal aimed at chick A in spite of the fact that B remained visible to A all of the time (Exp. 3 & Exp. 4).

FIGURE S2. The experimental arena, Related to STAR Methods

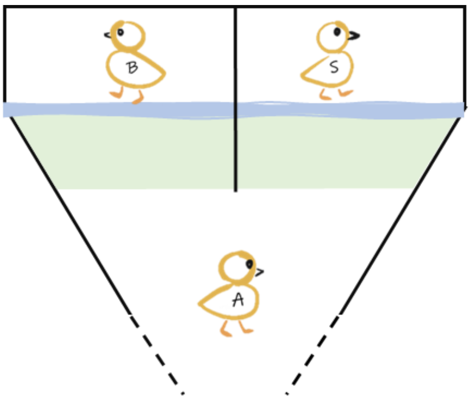

Fig. S2 Experimental arena. The conspecifics were confined in a rectangular area adjacent to the arena and separated from it by a one-way mirror. The tested chick (A) could see the

conspecifics but could not be seen by them. In one compartment was the cagemate of the tested chick (B, familiar chick); in the other compartment was an unknown chick matched for age and sec (S, stranger chick). B and S could not see each other as they were separated by an opaque partition. Such partition extended in the arena so as to create two separate choice areas (here highlighted in green), each associated with B or S, respectively.
